# The Dissector’s Manual

**Published:** 1884-01

**Authors:** 


**The Dissector's Manual.** By W. BRUCE CLARKE, M. A., M. B., Senior Demonstrator of Anatomy and Operative Surgeon at St. Bartholomew's Hospital, Examiner in Anatomy, University of Oxford, Surgeon to St. Peter's Hospital, etc., and C. B. LOCKWOOD, F. R. C. S. Illustrated with forty-nine engravings. H. C. Lea's Son & Co., Philadelphia, Pa.

This is one of the most useful aids to the student of anatomy that we have examined. It gives very full and clear directions how to find the various structures, what the student ought to see and be familiar with, and what he will not see unless special modes of preparation are employed to demonstrate them. The book is one which we would highly commend.

